# Defining metabolic migraine with a distinct subgroup of patients with suboptimal inflammatory and metabolic markers

**DOI:** 10.1038/s41598-023-28499-y

**Published:** 2023-03-07

**Authors:** Elena C. Gross, Niveditha Putananickal, Anna-Lena Orsini, Jean Schoenen, Dirk Fischer, Adrian Soto-Mota

**Affiliations:** 1 Division of Pediatric Neurology, University Children’s Hospital Basel (UKBB), University of Basel, Basel, Switzerland; 2 Division of Pediatric Neurology, University Children’s Hospital Basel (UKBB) & Neurology Department, University Hospital Basel (USB), University of Basel, Basel, Switzerland; 3 Headache Research Unit, Department of Neurology-Citadelle Hospital, University of Liège, Liège, Belgium; 4 Metabolic Diseases Research Unit, National Institute of Medical Sciences and Nutrition Salvador Zubirán (INCMNSZ), Tlalpan, Mexico; 5 School of Medicine, Tecnologico de Monterrey, Mexico City, Mexico

**Keywords:** Migraine, Biomarkers, Drug development

## Abstract

Emerging evidence suggest migraine is a response to cerebral energy deficiency or oxidative stress in the brain. Beta-hydroxybutyrate (BHB) is likely able to circumvent some of the meta-bolic abnormalities reported in migraine. Exogenous BHB was given to test this assumption and, in this post-hoc analysis, multiple metabolic biomarkers were identified to predict  clinical improvements. A randomized clinical trial, involving 41 patients with episodic migraine. Each treatment period was 12 weeks long, followed by eight weeks of washout phase / second run-in phase before entering the corresponding second treatment period. The primary endpoint was the number of migraine days in the last 4 weeks of treatment adjusted for baseline. BHB re-sponders were identified (those with at least a 3-day reduction in migraine days over placebo) and its predictors were evaluated using Akaike’s Information Criterion (AIC) stepwise boot-strapped analysis and logistic regression. Responder analysis showed that metabolic markers could identify a “metabolic migraine” subgroup, which responded to BHB with a 5.7 migraine days reduction compared to the placebo. This analysis provides further support for a “metabolic migraine” subtype. Additionally, these analyses identified low-cost and easily accessible biomarkers that could guide recruitment in future research on this subgroup of patients.

This study is part of the trial registration: ClinicalTrials.gov: NCT03132233, registered on 27.04.2017, https://clinicaltrials.gov/ct2/show/NCT03132233

## Introduction

Migraine is a common, complex and debilitating neurological disorder^1^, but its primary pathogenic mechanisms are not yet completely understood. Despite having been referred to as a “hypoglycemic headache” in 1935 already^2^, the focus of clinical and basic research in the last decades was primarily on (neuro-) vasculature, cerebral excitability, and neurotransmission. In recent years, metabolism and mitochondrial (dys-)function have regained interest. Various lines of evidence suggest migraine is—at least partially—a metabolic as much as a neurological disease, in which the migraine attack is a warning signal to increased oxidative stress and / or (cerebral) hypometabolism^3^.

Magnetic resonance spectroscopy (MRS) studies in migraineurs consistently show decreased ATP levels or hypometabolism^4–15^. Mitochondrial function and oxidative stress markers have also been shown to be abnormal in higher-frequency migraine^16^. Additional support comes from early studies demonstrating metabolic changes induced by fasting, glucose or insulin administration, which were shown to be able to even trigger migraine attacks in susceptible patients^16–22^.

Several nutraceuticals^23^, such as riboflavin at high dose (200–400 mg/day)^30–36^; coenzyme Q10 (400 mg capsules or 300 mg liquid suspension)^24–29^, magnesium^37^ and alpha-lipoic acid (600 mg)^38–40^ have shown to prevent migraine attacks also suggesting a link between migraine and metabolism/or mitochondrial functioning.

Oxidative stress seems to be the common denominator of most migraine triggers^41,42^ and apart from clearly “metabolic” triggers (such as physical exercise, fasting, and stress), many of the seemingly unrelated triggers (like ovarian hormone changes, alcohol, weather changes, intense light, and strong odors) can negatively impact mitochondrial metabolism and/or oxidative stress (see reviews^3,41^). Mechanistically, nitrosative, oxidative, and electrophilic stress can activate transient receptor potential channels, expressed in meningeal nociceptive nerve terminals^43,44^, thereby providing a mechanism by which known migraine trigger factors which increase oxidative stress could lead to migraine pain.

Metabolic approaches to migraine prevention, such as a ketogenic diet (KD), which to some extent mimics the state of fasting, have been shown to be migraine protective^44–49^. The KD was developed over 100 years ago, after the observation that prolonged fasting has antiepileptic properties^50^. Like fasting, it promotes the hepatic production of ketone bodies (KBs). Recently the KD has received renewed interest, due to the observation that KBs could be beneficial for a variety of other neurological disorders as^51–53^ all brain cells have the capacity to use KBs as respiratory substrates^54^.

Out of the three physiologically relevant KBs β-hydroxybutyrate (BHB) constitutes up to 70%^55^ and acts also as a signaling molecule^56^. Consequently, it has the potential to positively influence other pathways commonly believed to be part of migraine pathophysiology^57^.

In complex and heterogenous diseases such as migraine, a therapy that can simultaneously target multiple possible pathogenic pathways seems advantageous and elevated KB levels have been shown to be well tolerated for extended periods of time, even up to several years^47,58–70^. However, a very strict KD, may be difficult to adhere to longer-term.

Our research group wanted to examine whether exogenously raised KBs would also be able to attenuate migraine frequency, if ingested daily, and carried out the first RCT exploring the effect of BHB as a prophylactic agent in episodic migraine patients^71^ where a non-statistically significant reduction of 1.9 migraine days over placebo was documented, however, some patients clearly reduced more days than other. In line with the already outline evidence supporting the existence of a “metabolic migraine subgroup”, we aimed to evaluate if metabolic health markers could identify patients responded to BHB supplementation.

## Materials and methods

### Trial design

The trial conducted was a double-blind, randomized, placebo-controlled trial with a crossover design with 41 migraine patients meeting the ICHD-3 (International Classification of Headache Disorders version 3 Beta) Classification criteria^73^. The trial was registered at ClinicalTrials.gov (NCT03132233), approved by the local ethics committee Swissethics (EKNZ 2015-304) and the National Swiss Drug Agency (2016DR2109). The detailed methods can be found in the published study protocol^72^. In brief, the trial consisted of a four-week run-in period followed by randomization. Then a first treatment period of 12 weeks, followed by a washout period of 4 weeks. Afterward, a second run-in phase of 4 weeks and finished with the second treatment period of 12 weeks.

### Study medication

The investigational medicinal product (IMP) used in this clinical trial was 9 gr of D-BHB (from 18 gr racemic BHB) in powdered calcium (Ca2 +)–magnesium (Mg2 +)–salt form (Ca–Mg–BHB) divided into three servings per day. The mineral load determined the maximal IMP dose. The placebo group received sachets containing Mannitol. 

### Clinical measures

At pre- and post- intervention visits the following assessments were additionally conducted: Migraine Disability Test (MIDAS)^74^, Headache Impact Test, version 6 (HIT-6)^75^ and blood draw for biomarker and safety analysis (albumin, Calcium, cortisol, alanine aminotransferase, pancreatic-amylase, alkaline phosphatase, aspartate aminotransferase, beta-hydroxybutyrate, bilirubin, creatine kinase, Chloride, Cholesterol, Cholesterol Quotient, Cortisol basal, high sensitivity-C reactive protein, globulin, fasting glucose, gamma-glutamyl transferase, glomerular filtration rate, uric acid, HbA1c, High density lipoprotein, urea, Potassium, creatinine, lactate plasma, lactate dehydrogenase, low-density lipoprotein (calculated with Friedewald’s equation), Magnesium, Sodium, Phosphate, total protein, triglycerides, leukocyte count, erythrocytes, hemoglobin, hematocrit, mean corpuscular volume, platelets, T3, T4, Insulin and thyroid stimulating hormone (TSH). All blood samples were taken after an overnight fast between 8 and 11 am and all markers were considered in the responder analysis. Further details on data collection are provided in the published study protocol^72^.

### Statistical analysis

Data wrangling and statistical analyses for this purpose were performed using R version 4.0.3. and the packages: tidyverse, readxl, performance, tableone, gtools, MASS, bootStepAIC, lmtest, rpart and car. When relevant, data and all linear model residuals were tested for normality using stats::shapiro.test. A Friedman Test was used to analyze pharmacokinetics data and differences between responders and non-responders were analyzed using Mann–Whitney and Kruskal–Wallis’s rank sum tests. Baseline vs follow-up metabolite changes were analyzed using Wilcoxon tests.

To identify factors associated with positively responding to KBs supplementation, we evaluated the relevance of different combinations of independent predictors according with the explanatory capacity of each model Akaike’s Information Criterion (AIC), the consistency of their coefficient signs, and the consistency of their statistical relevance. This procedure was performed via a bootstrap AIC consistency diagnosis in which 100 independent samples drawn at random from the dataset using *bootStepAIC::boot.stepAIC*. To avoid collinearity, we analyzed potential models by grouping blood markers according with their corresponding physiological system (thyroid markers, liver function markers, blood cells markers, etc.) to identify the best predictor from each system and test its predictive contribution to different potential models. For evaluating the all-around performance of the combined models for predicting BHB response, we used *performance::compare_performance* which allows for simultaneously comparing AIC, Bayes Information Criterion (BIC), Root mean squared error (RMSE), and Tjur’s R^2^. Linear assumptions of the models we corroborated using *performance::check_model*.

Finally, we used supervised machine-learning regression trees to identify potentially useful cutoffs for relevant predictors using *rpart::rpart*.

### Local ethical approval and consent to participate

All participants provided informed consent to participate in the trial. Ethical approval was granted by Swissethics, EKNZ PB 2016-00497. Also, all methods were performed in accordance with the relevant guidelines and regulations.

## Results

### Study population

A total of 9 out of 32 patients (28.13%) met our conservative criteria for BHB treatment response, ranging from 3- to 12-day reduction in migraine days from baseline compared to placebo. Treatment responders had an average of 5.78 (SD = 2.63) less migraine days compared to placebo.

**Figure 1:**
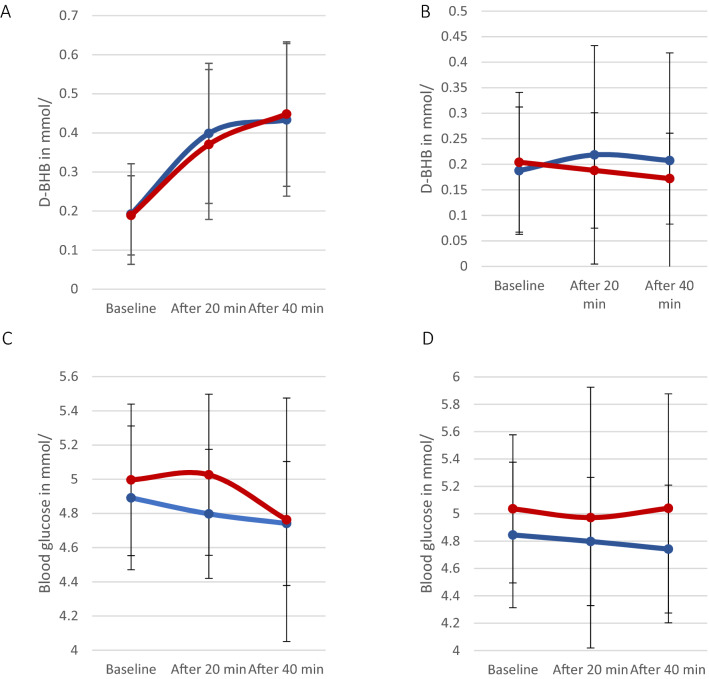
Blood BHB and glucose levels with IMP (panels (**A**) and (**C**)) placebo (panels (**B**) and (**D**)) in non-responders (blue) & responders (red). Error bars depict the standard deviation (SD).

To evaluate is pharmacokinetic differences were likely responsible for the differences in therapeutic success, a Friedman test was conducted between responders and non-responders for glucose and BHB (Fig. 1).

Table 1 describes the distribution and demographic and metabolic differences between responders and not responders at baseline.

**Table 1:** Differences in metabolites between responders and non-responders at baseline.

	Non-responder (n = 23)					Responders (n = 9)					p value
	Mean	Std. dev	p50	p25	p75	Mean	Std. dev	p50	p25	p75	
HS-CRP	1.7	2.0	1.4	0.5	1.7	3	3.37	2.4	0.8	3	< 0.001
TSH	2	0.8	1.9	1.3	2.5	2.5	2.53	1.9	1.4	3	0.182
Albumin	40.2	3.0	40	38	42	39.8	2.59	40	39	41	0.778
ALT	23.4	10.0	19	15	25	16.2	4.63	15	13	19	< 0.001
BHB	0.2	0.2	0.2	0.1	0.2	0.2	0.12	0.2	0.1	0.3	0.822
Cortisol	363.9	200.0	327	263	363.9	266.3	89.39	264	211	303	< 0.001
Glucose	4.9	0.5	4.8	4.6	5.1	5	0.48	5	4.7	5.4	0.056
HbA1c	5	0.3	5	4.8	5.2	5.2	0.27	5.1	5	5.3	< 0.001
HDL	1.6	0.3	1.6	1.4	1.8	1.6	0.2	1.6	1.5	1.8	0.338
Lactate	1.2	0.7	1	0.8	1.4	1.4	1.15	0.9	0.7	1.5	0.894
LDH	173.7	20.0	171	156.8	188.2	161.7	33.33	156	141	172	< 0.001
LDL	2.3	0.8	2.3	1.7	2.9	2.6	0.85	2.3	1.9	2.9	0.211
Mg	0.8	0.1	0.8	0.8	0.9	0.8	0.06	0.8	0.8	0.9	0.803
Na	140.4	2.0	140	139	142	140.3	2.21	140	139	142	0.866
Pi	1.1	0.2	1.1	1	1.2	1	0.17	1	0.9	1.1	< 0.001
Triglycerides	1	0.4	0.8	0.6	1.2	0.9	0.28	0.8	0.7	1	0.440
FT3	4.9	0.8	4.9	4.4	5.3	4.6	0.75	4.4	4.2	4.8	< 0.001
FT4	15.8	2.0	15.8	14.7	16.8	16	3.36	15.3	14.1	16.4	0.373
Insulin	9.2	5.0	8.6	5.9	10.9	8.7	2.9	8.3	6.8	10.2	0.888
Age	36.4	10	33	28	45	44	10	45	35	53	0.08
BMI	23.4	4.00	22.4	20.8	25.1	24.5	4	23.9	21.2	25.4	0.52
Sex	*Female*	27 (87.1%)	*Male*	4 (12.9%)		*Female*	8 (88.9%)	Male	1 (11.1%)		0.06

P-values were obtained using *tableone::CreateTableOne* which uses Kruskal–Wallis’s rank sum tests for non-parametric hypothesis testing.

**Figure 2:**
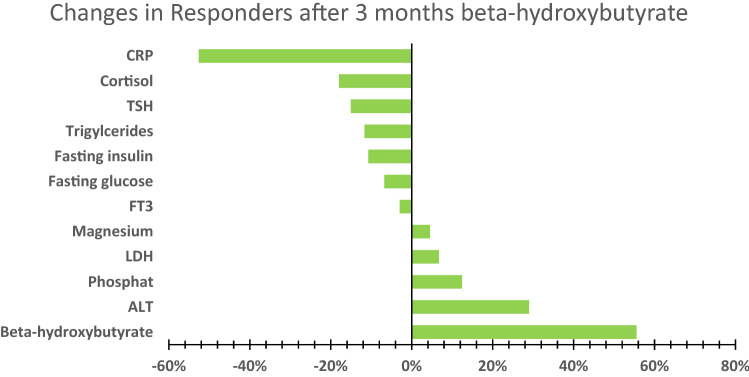
Metabolite changes in responders after 3 months with the IMP in percent difference from baseline values. (n = 9).

After 3 months of BHB supplementation many of these markers changed into the direction of the non-responder levels. TSH dropped by 15%, triglycerides by 12%, fasting glucose by 7%, hs-CRP by 53% and endogenous BHB levels increased by 56% in the responder group. In addition, fasting insulin dropped by 11% and cortisol levels by 18%. Furthermore, ALT increased by 29%, phosphate by 12%, LDH by 7% and magnesium by 5% (see Fig. 2). Only the change in CRP was statistically significant (p = 0.002) after using Wilcoxon’s hypothesis testing.

After comparing multiple logistic regression models with 100 bootstrapped samples from all available measurements, we concluded that, because of their cost, availability and Beta coefficient consistency, C-reactive protein, Phosphorus and HbA1c are the most useful predictors of BHB supplementation responsiveness. All were statistically significant below the 0.05 threshold in > 95% of the bootstrap simulations and the sign of their estimate was consistent 100% of the times. Only variables with a 100% coefficient sign consistency were selected for the candidate models and, to avoid collinearity and physiological “redundance”, only one predictor per system was used. Table 2 summarizes the all-around performance of the candidate models according with their AIC, BIC, RMSE and R^2^.

**Table 2:** Performance comparison of candidate logistic regression models.

Model	AIC	BIC	R2	RMSE
All metabolites	113.6	207.7	0.78	0.19
Pi + HS − CRP + FT3 + HbA1c	209.2	226.0	0.18	0.39
Pi + HS − CRP + FT3 + ALT	192.3	209.2	0.26	0.37
Pi + HS − CRP + HbA1c	218.3	231.7	0.13	0.40
Pi + HS − CRP + FT3 + ALT + HbA1c + LDH	173.3	196.8	0.35	0.34

Performance of logistic regression models for the outcome “responder”.

*AIC* Akaike’s information criterion, *BIC* Bayes information criterion, *R*^*2*^ Tjur’s R^2^, *RMSE* root mean square error.

The model including Pi + HS − CRP + FT3 + ALT accounts for 26% of the variance using only 4 predictors. Adding 2 more predictors; HbA1c and LDH, improved the explained variance and accounts for almost half of the variance explained by the model using all metabolites. After identifying potentially useful markers, we used a regression tree to then identify their potentially useful cut-off points.

**Figure 3:**
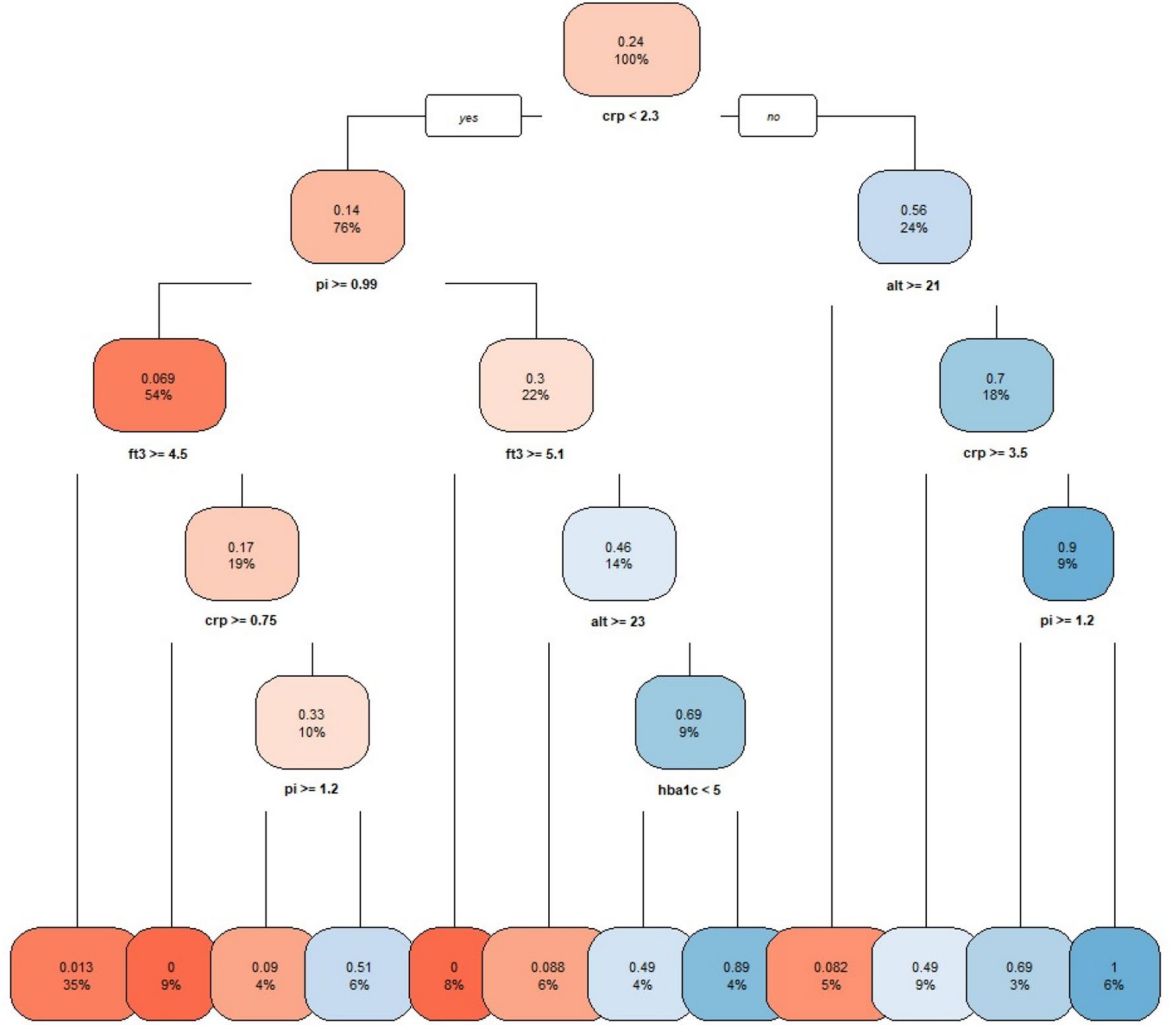
Regression tree of using Pi + LDH + HS − CRP + FT3 + ALT + HbA1c and the R function rpart::rpart. Color blue = ” responders” and color red = ” non-responders”.

As suggested by Fig. 3, using these cut-off points, the least and apparently most useful markers for identifying whether a person belongs to the responder group were inorganic phosphorous, hs-CRP (a marker for inflammation) and Hba1C (long-term blood sugar). This model’s linear assumptions were corroborated (Supplemental Information).

## Discussion

Classifying sub-groups of migraineurs based on objective biomarkers is essential for improving clinical study designs, developing novel treatments and ultimately, improving clinical care. Hereby, we documented metabolic markers in BHB supplementation responders differed from those who did not respond. To our knowledge, this is the first work proposing blood biomarkers for predicting treatment response to migraine prophylaxis and could help pave the way not only for testing future anti-inflammatory/metabolic interventions, but also for reassessing already existing solutions^76^.

However, there were several limitations to this analysis. At trial onset, only the racemic BHB was available, which has ½ of the potency of the bioidentical D- BHB^77^. In addition, the dose of D-BHB (9 g per day) was low compared to the 185 g of KBs produced by the liver during fasting^78^. The upper limit was determined by the mineral’s upper daily intake requirements that the BHB was bound to. Due to these two factors, the potency of the current formulation was so low that nutritional ketosis (> 0.5 mmol/l BHB) was never reached^79^. Furthermore, mannitol was used as a placebo, but it may be not a be an ideal placebo because it shares one migraine relevant mechanism as it increases brain tissue oxygenation^80,81^. Not surprisingly, we identified 4 responding to mannitol.

Despite the trial’s limitations, we could find potentially useful and easily available predictors (and their potentially useful cut-offs) for identifying whether a person belongs to the responder group or not based on the independent markers of inorganic phosphorous, HS-CRP (a marker for inflammation) and Hba1C (long-term blood sugar) and this result is highly significant—despite our small sample size but should be prospectively validated in future studies.

In contrast, several markers of metabolism and inflammation were worse in the BHB responder group while no pharmacokinetic differences were found. Additionally, most of these markers improved or started looking more like the non-responder group after the intervention. Most notably hs-CRP (inflammation) more than halved and this change was significant, despite the small sample size (n = 9), thus, it is likely the rest of the paired analyses were underpowered. Together, these findings suggest that responders were responding because of their baseline pathophysiological differences and not because they were exposed to higher doses of BHB.

While the connection to energy metabolism with glucose, insulin, KBs, HbA1c and triglycerides seems evident, the other markers also have a strong connection to metabolism. Hs-CRP has been found to be a marker of impaired energy metabolism, in addition to a marker of acute systemic inflammation^82^, the thyroid is well known for its signalling role in energy homeostasis and energy metabolism^83^, and ALT has been shown to play a role in metabolic disease^84^. The exact mechanisms by which KB improve or prevent migraine can be multiple and additive ranging from restoring energy utilisation to ameliorating inflammation^85^.

It is necessary to mention we are not the first ones to find an association between treatment response to a metabolic migraine therapeutic and a biomarker. Over a decade ago the therapeutic response to high-dose riboflavin was shown to be associated with specific mitochondrial DNA (mtDNA) haplogroups (non- H mitochondrial DNA haplotypes)^36^, which is also indicative of a “metabolic migraine subgroup”. However, mtDNA haplogroups are, however, not as easily identified as the common three laboratory markers that we propose. These biomarkers could be used to guide the inclusion criteria of future clinical trials and aid the selection of “metabolic migraineurs” for future trials.

We should highlight that the utility of these individual predictors is context dependant and we provided data for cost–benefit assessments. For example, as shown in Table 2, adding FT3 and ALT to Pi + HS − CRP + HbA1c doubles the R^2^ but would likely complicate recruitment as there are not many point-of-care options for measuring them. Importantly, the identified cut-offs in this work need to be validated prospectively but, the fact that they are already close to those currently used in clinical practice suggest.

Finally, it is possible that these potentially relevant metabolic predictors are not migraine specific and could be useful for studies on other illnesses for which ketosis has been hypothesized to be beneficial such as diabetes, heart failure and epilepsy.

## Conclusion

This study provides further support for a distinct “inflammatory/ metabolic migraine” subgroup with unique metabolic and inflammatory signatures. Three easy to measure blood markers (hs-CRP, HbA1c and phosphorus) could assist personalized metabolic migraine treatments and prophylactic interventions.

## Supplementary Information

Supplementary Information.

## Data Availability

The datasets generated and/or analysed during the current study are not publicly available due to parts of the data set still being analysed at present but are available from the corresponding author on reasonable request.

## Abbreviations

AE: Adverse event
ALT: Alanine aminotransferase
AST: Aspartate aminotransferase
AIC: Akaike’s information criterion
BIC: Bayes information criterion
RMSE: Root mean square error
ATP: Adenosine triphosphate
BHB: Beta-hydroxybutyrate
BMI: Body mass index
Ca: Calcium
CI: Confidence interval
HS-CRP: High-sensitivity C-reactive protein
CTU: Clinical trial unit
HbA1c: Glycosylated hemoglobin
GMP: Good manufacturing practice
ICHD-3: International classification of headache disorders version 3
IMP: Investigational medicinal product
ITT: Intention to treat
HDL: High density lipoprotein
HIT: Headache impact test
IQR: Interquartile range
KB: Ketone bodies
KD: Ketogenic diet
LDH: Lactate dehydrogenase
LDL: Low density lipoprotein
MIDAS: Migraine disability assessment
Mg: Magnesium
31P-MRS: Phosphorus‐31 magnetic resonance spectroscopy
Na: Sodium
NSAIDs: Non-steroidal anti-inflammatory drugs
Pi: Phosphorous
PP: Per protocol
RCT: Randomized clinical trial
ROS: Reactive oxygen species
TSH: Thyroid stimulating hormone
USB: University hospital basel
VAS: Visual analog scale
